# Association between Open-Angle Glaucoma and the Risks of Alzheimer’s and Parkinson’s Diseases in South Korea: A 10-year Nationwide Cohort Study

**DOI:** 10.1038/s41598-018-29557-6

**Published:** 2018-07-24

**Authors:** Jong Youn Moon, Hyung Jun Kim, Yoon Hyung Park, Tae Kwann Park, Eun-Cheol Park, Chan Yun Kim, Si Hyung Lee

**Affiliations:** 1 Department of Preventive Medicine, Soonchunhyang University, College of Medicine, Cheonan, Republic of Korea; 2 Institute of Health Services Research, Yonsei University, College of Medicine, Seoul, Korea; 3 Department of Neurology, Soonchunhyang University Hospital Bucheon, Bucheon, Republic of Korea; 4 Department of Ophthalmology, Soonchunhyang University Hospital Bucheon, Bucheon, Republic of Korea; 5 Institute of Vision Research, Department of Ophthalmology, Severance Hospital, Yonsei University, College of Medicine, Seoul, Korea

## Abstract

We aimed to investigate the risks of Alzheimer’s (AD) and Parkinson’s disease (PD) in the 10 years following diagnosis of open-angle glaucoma (OAG) using a nationwide cohort. This propensity score-matched retrospective cohort study included 1,025,340 subjects from the Korean National Health Insurance Service National Sample Cohort database. The OAG group (n = 1,469) included patients who were initially diagnosed with OAG between 2004 and 2007, and the subjects in the comparison group were matched in a 1:5 ratio using propensity scores. Cox regression analyses were performed to investigate the risks of developing AD or PD. The diagnosis of OAG was significantly associated with an increased incidence of AD (hazard ratio [HR] = 1.403, 95% confidence interval [CI] 1.180–1.669, *p* < 0.001), but not PD (HR = 0.995, 95% CI 0.620–1.595, *p* = 0.983) after adjusting for possible confounding factors. In subgroup analyses, participants with OAG aged ≥65 years were more likely to develop AD compared with those aged <65 years, and female OAG patients had a greater risk of developing AD than males. Patients diagnosed with OAG have a higher risk of developing AD, but not PD, and the risk differed according to age and sex.

## Introduction

Open-angle glaucoma (OAG) is a leading cause of irreversible blindness and is characterized by chronic progressive glaucomatous optic neuropathy with corresponding visual field defects^1,2^. Retinal ganglion cells (RGCs), the main target of glaucomatous damage, are central nervous system neurons located in the inner layer of the retina that convey information from the retina to the brain. On developing glaucoma, injuries occur in optic nerve axons, leading to progression of RGC neurodegeneration^3,4^. It is widely accepted that intraocular pressure (IOP) plays a crucial role in the development and progression of OAG, and several non-IOP factors have been reported to be associated with OAG pathogenesis^5–8^. However, there are still many mysteries to unravel to determine the mechanisms involved in RGC damage in glaucoma patients.

Alzheimer’s disease (AD) and Parkinson’s disease (PD) are the two most common neurodegenerative disorders in the elderly^9^. In AD, neurons and synapses are lost in the cerebral cortex, in the presence of extracellular amyloid-β plaques (Aβ) and neurofibrillary tangles that consist of phosphorylated Tau protein, leading to progressive memory loss, behavioral disturbances, personality changes, and cognitive impairment^10,11^. PD is a chronic, progressive movement disorder in which patients show slow movement, difficulty walking, rigidity, and tremor, caused by the loss of dopaminergic neurons in the substantia nigra^12,13^. It is related to alpha-synuclein, the major biochemical component of Lewy bodies^14^. Neurodegenerative disease, *i*.*e*., the progressive loss of neuron structure or function, including AD and PD, is currently recognized as a major public health issue with the aging global population^15,16^. There is increasing evidence of neurodegenerative lesions found in AD are also observed in the optic nerve, lateral geniculate nucleus, and visual cortex in glaucoma patients, suggesting that OAG is a neurodegenerative disease of the eye^17–24^. Previous experimental and clinical studies have shown close associations between OAG and PD^25–27^. These two neurodegenerative diseases, AD and PD, and glaucoma share several biological features, such as being chronic, slowly progressive disorders with an age-related incidence and similar mechanisms of cell injury. Although there are strong associations between OAG and AD or PD, only a few epidemiological studies have been conducted with inconsistent results, leaving the relationship between the conditions inconclusive^28–33^.

In light of this, we investigated the risks of developing AD and PD after an initial diagnosis of OAG, and compared the risk of each neurodegenerative disease during the 10 years following diagnosis of OAG using a representative South Korean sample of approximately 1 million adults from the National Health Insurance Service-National Sample Cohort 2002–2013 (NHIS-NSC 2002–2013).

## Results

Table 1 shows the baseline characteristics of the study population. The study ultimately enrolled 1,469 patients with OAG and 7,345 individuals for the comparison group. There was a significant difference in AD prevalence between the OAG group and the comparison group (*p* < 0.001). Hypertension (*p* < 0.001), diabetes mellitus (*p* < 0.001), hyperlipidemia (*p* < 0.001), and ischemic stroke (*p* = 0.003) were more prevalent in the OAG group than in the comparison group. There were no significant differences in the other parameters used for matching, which included sex, age group, income, residential area, Charlson comorbidity index (CCI), and the year of enrollment. When subjects were grouped according to the diagnosis of each neurodegenerative disease, subsequent AD developed in 742 subjects and subsequent PD developed in 118 subjects during the median follow-up period of 6.99 years. Subjects with AD and PD had higher proportions of female, old age, high income, and high CCI than patients without these diseases (Table 2).

**Table 1:** Demographic characteristics of study population.

	Patient without OAG		Patient with OAG		P-value	Total
	N	%	N	%		N
Total	7,345	100.0%	1,469	100.0%		8,814
**Presence of AD or PD**						
No	6,682	91.0%	1,272	86.6%	<0.001	7,954
AD	567	7.7%	175	11.9%		742
PD	96	1.3%	22	1.5%		118
**Hypertension**						
No	2,615	35.6%	446	30.4%	<0.001	3,061
Yes	4,730	64.4%	1,023	69.6%		5,753
Diabetes						
No	3,112	42.4%	539	36.7%	<0.001	3,651
Yes	4,233	57.6%	930	63.3%		5,163
**Hyperlipidemia**						
No	2,582	35.2%	429	29.2%	<0.001	3,011
Yes	4,763	64.8%	1,040	70.8%		5,803
Ischemic stroke						
No	5,836	79.5%	1,117	76.0%	0.003	6,953
Yes	1,509	20.5%	352	24.0%		1,861
**Variables for matching**						
**Sex**						
Male	3,886	52.9%	781	53.2%	0.856	4,667
Female	3,459	47.1%	688	46.8%		4,147
**Age group**						
≤49	1,852	25.2%	372	25.3%	0.958	2,224
50–59	1,447	19.7%	287	19.6%		1,734
60–69	2,239	30.5%	436	29.7%		2,675
70–79	1,477	20.1%	306	20.8%		1,783
≥80	330	4.5%	68	4.6%		398
**Income**						
High	3,861	52.6%	759	51.7%	0.684	4,620
Milddle	2,142	29.2%	428	29.1%		2,570
Low	1,342	18.3%	282	19.2%		1,624
**Residential area**						
Metropolitan	514	7.0%	106	7.2%	0.944	620
City	3,299	44.9%	655	44.6%		3,954
Rural	3,532	48.1%	708	48.2%		4,240
**CCI**						
≤3	5,891	80.2%	1,176	80.1%	0.895	7,067
>3	1,454	19.8%	293	19.9%		1,747
**Year**						
2004	1,435	19.5%	287	19.5%	1.000	1,722
2005	1,495	20.4%	299	20.4%		1,794
2006	1,395	19.0%	279	19.0%		1,674
2007	1,355	18.4%	271	18.4%		1,626
2008	1,665	22.7%	333	22.7%		1,998

OAG = open angle glaucoma; AD = Alzheimer’s disease; PD = Parkinson’s disease; CCI = Charlson comorbidity index.

**Table 2:** Characteristics according to the presence of Alzheimer’s disease or Parkinson’s disease.

	Patients without AD or PD		AD		PD		Total
Total	7954	(90.2%)	742	(8.4%)	118	(1.3%)	8,814
**OAG**							
No	6,682	(84.0%)	567	(76.4%)	96	(81.4%)	7,345
Yes	1,272	(16.0%)	175	(23.6%)	22	(18.6%)	1,469
**Hypertension**							
No	2,988	(37.6%)	62	(8.4%)	11	(9.3%)	3,061
Yes	4,966	(62.4%)	680	(91.6%)	107	(90.7%)	5,753
**Diabetes**							
No	3,506	(44.1%)	119	(16.0%)	26	(22.0%)	3,651
Yes	4,448	(55.9%)	623	(84.0%)	92	(78.0%)	5,163
**Hyperlipidemia**							
No	2,873	(36.1%)	114	(15.4%)	24	(20.3%)	3,011
Yes	5,081	(63.9%)	628	(84.6%)	94	(79.7%)	5,803
**Ischemic stroke**							
No	6,620	(83.2%)	279	(37.6%)	54	(45.8%)	6,953
Yes	1,334	(16.8%)	463	(62.4%)	64	(54.2%)	1,861
**Variables for matching**							
**Sex**							
Male	4,298	(54.0%)	318	(42.9%)	51	(43.2%)	4,667
Female	3,656	(46.0%)	424	(57.1%)	67	(56.8%)	4,147
**Age group**							
≤49	2,221	(27.9%)	1	(0.1%)	2	(1.7%)	2,224
50–59	1,690	(21.3%)	36	(4.9%)	8	(6.8%)	1,734
60–69	2,423	(30.5%)	201	(27.1%)	51	(43.2%)	2,675
70–79	1,337	(16.8%)	399	(53.8%)	47	(39.8%)	1,783
≥80	283	(3.6%)	105	(14.2%)	10	(8.5%)	398
**Income**							
High	4,095	(51.5%)	452	(60.9%)	73	(61.9%)	4,620
Milddle	2,395	(30.1%)	150	(20.2%)	25	(21.2%)	2,570
Low	1,464	(18.4%)	140	(18.9%)	20	(16.9%)	1,624
**Residential area**							
Metropolitan	558	(7.0%)	58	(7.8%)	4	(3.4%)	620
City	3,597	(45.2%)	304	(41.0%)	53	(44.9%)	3,954
Rural	3,799	(47.8%)	380	(51.2%)	61	(51.7%)	4,240
**CCI**							
≤3	6,655	(83.7%)	350	(47.2%)	62	(52.5%)	7,067
>3	1,299	(16.3%)	392	(52.8%)	56	(47.5%)	1,747
**Year**							
2004	1,524	(19.2%)	173	(23.3%)	25	(21.2%)	1,722
2005	1,594	(20.0%)	177	(23.9%)	23	(19.5%)	1,794
2006	1,524	(19.2%)	128	(17.3%)	22	(18.6%)	1,674
2007	1,483	(18.6%)	121	(16.3%)	22	(18.6%)	1,626
2008	1,829	(23.0%)	143	(19.3%)	26	(22.0%)	1,998

OAG = open angle glaucoma; AD = Alzheimer’s disease; PD = Parkinson’s disease; CCI = Charlson comorbidity index.

The risk of developing subsequent AD or PD during the 10-year follow-up period was evaluated using multivariate Cox hazard regression analyses with three different models, crude model, model 1 hypertension, diabetes mellitus, hyperlipidemia and ischemic stroke) and model 2 (adjusted for age, sex, residential area, income, CCI, hypertension, diabetes mellitus, hyperlipidemia and ischemic stroke). All three models revealed that the risk of developing subsequent AD during the 10-year follow-up period was significantly higher in the OAG group than in the comparison group (Crude: hazard ratio [HR] = 1.623, 95% confidence interval [CI] 1.369–1.924, *p* < 0.001; model 1: HR = 1.421, 95% CI 1.199–1.684, *p* < 0.001; model 2: HR = 1.403, 95% CI 1.180–1.669, *p* < 0.001) (Table 3). In model 1, hypertension, diabetes mellitus, ischemic stroke were also associated with an increased risk of AD development (Supplementary Table 1), while in model 2, old age, female gender, ischemic stroke and a high CCI score were significantly associated with an increased risk of developing AD (Supplementary Table 2). In comparison, the risk of developing PD in the OAG group was not significant compared with the comparison group in all three models (Crude: HR = 1.115, 95% CI 0.696–1.788, *p* = 0.650; model 1: HR = 0.984, 95% CI 0.612–1.582, *p* = 0.946; model 2: HR = 0.995, 95% CI 0.620–1.595, *p* = 0.983), and there were disparities in the development of PD according to hypertension and ischemic stroke in model 1, and ischemic stroke and subject age in model 2. (Supplementary Tables 1 and 2).

**Table 3:** Hazard ratios for Alzheimer’s disease or Parkinson’s disease in multivariable Cox regression analysis.

Outcome		Crude		Model 1^1^		Model 2^2^	
		Hazard ratio (95% CI)	p-value	Hazard ratio (95% CI)	p-value	Hazard ratio (95% CI)	p-value
AD	No OAG	1.000 (reference)		1.000 (reference)		1.000 (reference)	
	OAG	1.623 (1.369–1.924)	<0.001	1.421 (1.199–1.684)	<0.001	1.403 (1.180–1.669)	<0.001
PD	No OAG	1.000 (reference)		1.000 (reference)		1.000 (reference)	
	OAG	1.115 (0.696–1.788)	0.650	0.984 (0.612–1.582)	0.946	0.995 (0.620–1.595)	0.983

^1^Adjusted for hypertension, diabetes mellitus, hyperlipidemia and ischemic stroke.

^2^Adjusted for age, sex, residential area, income, Charlson comorbidity index, hypertension, diabetes mellitus, hyperlipidemia and ischemic stroke.

OAG = open angle glaucoma; AD = Alzheimer’s disease; PD = Parkinson’s disease.

**Figure 1:**
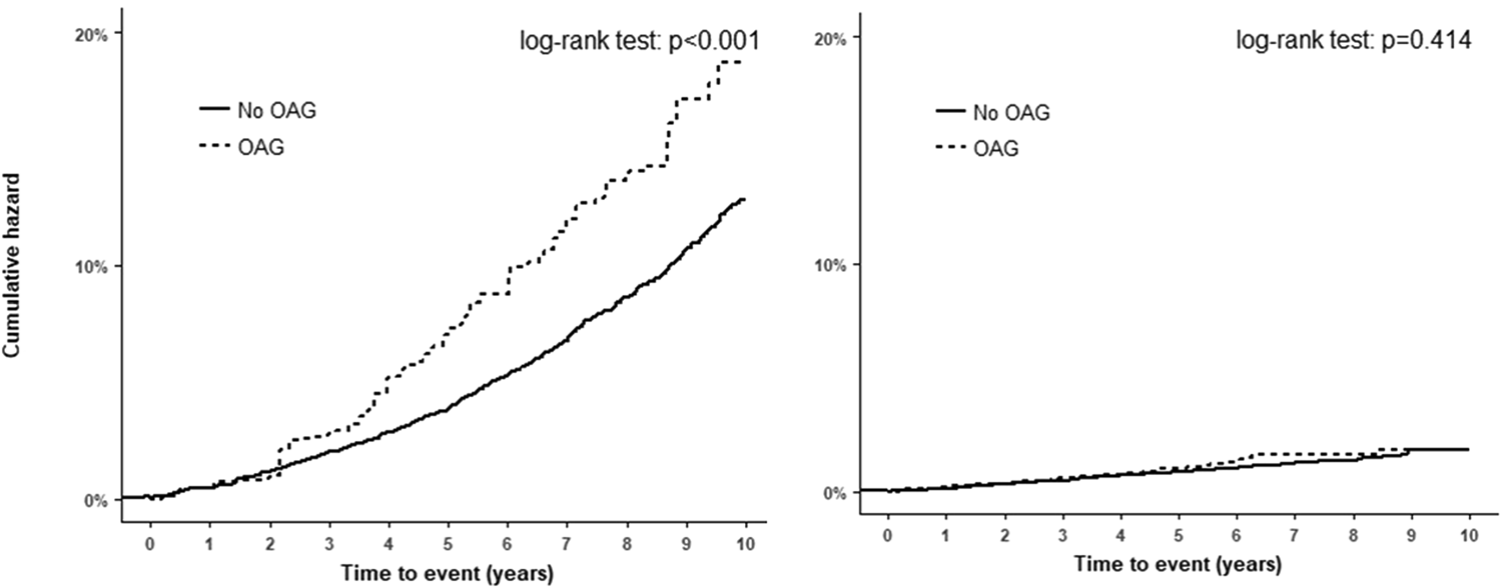
Cumulative hazards of (**a**) Alzheimer’s disease and (**b**) Parkinson’s disease.

The Kaplan–Meier survival curves showed that the risk of developing AD among OAG patients was higher than in the comparison group (log-rank test, *p* < 0.001, (Fig. 1a), whereas no significant difference in the cumulative PD incidence was observed between the OAG and comparison groups (log-rank test, *p* = 0.414, Fig. 1b).

**Figure 2:**
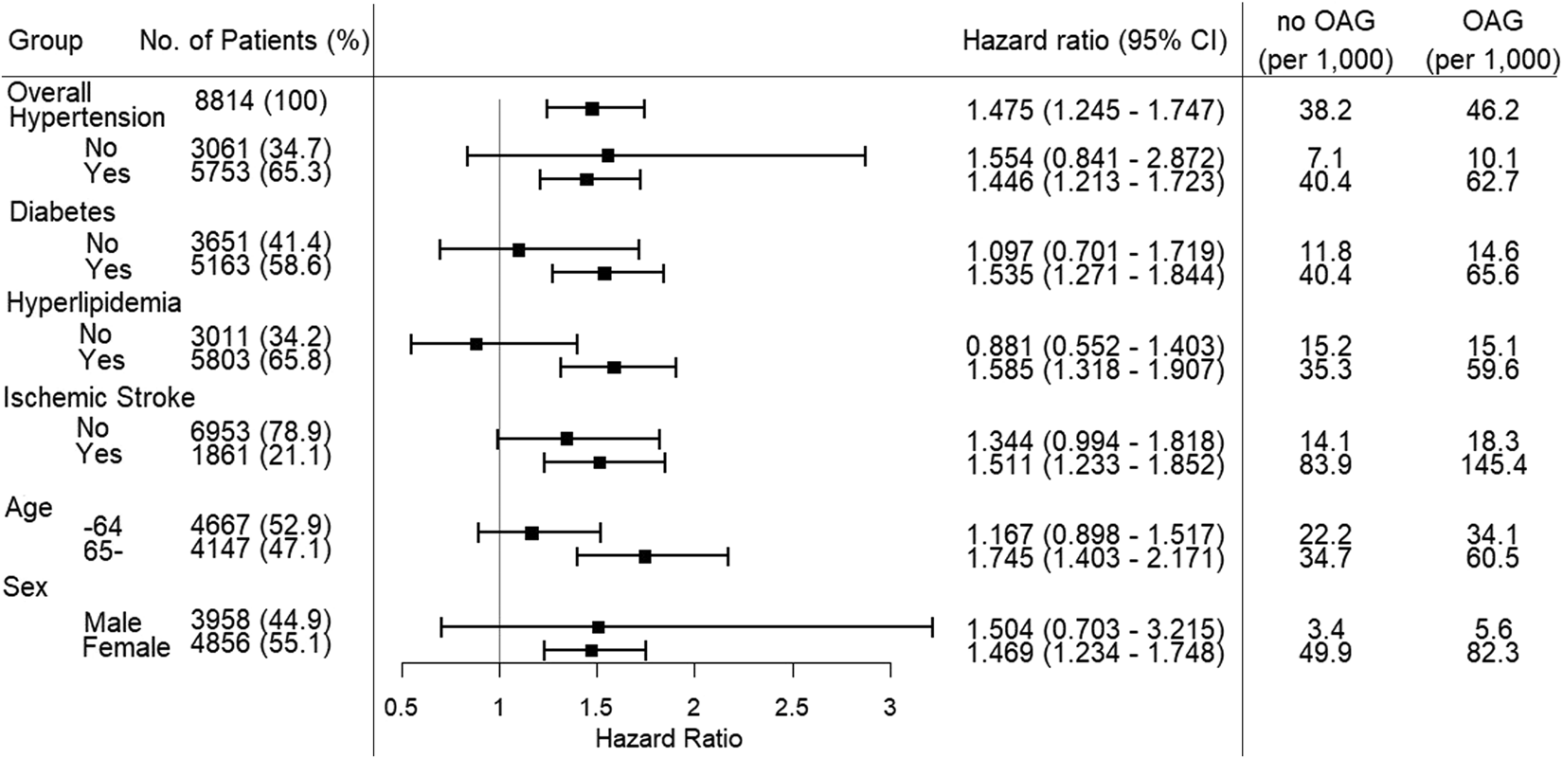
Subgroup analysis of Alzheimer’s disease based on multivariate Cox regression.

The incidence of AD per 1,000 person-years for the OAG and comparison groups was 38.2% and 46.2%, respectively (Fig. 2). In subgroup analyses based on multivariate Cox regression, the adjusted HR of AD in the OAG patients with diabetes mellitus was greater (HR = 1.535) than that in the subjects without diabetes mellitus (HR = 1.097). The risk of developing AD in the OAG group was higher in those with hyperlipidemia or ischemic stroke (HR = 1.585 and HR = 1.511, respectively) compared with those without hyperlipidemia or ischemic stroke (HR = 0.881 and HR = 1.344, respectively). The risk of developing AD in patients with OAG was higher in the older subgroup (age ≥ 65 years; HR = 1.745) than that in the younger subgroup (age < 65 years; HR = 1.167). Subgroups for hypertension and sex had a similar HR to that of the entire cohort regardless of the presence of the disease or gender.

## Discussion

Many studies have suggested that glaucoma is an age-related neurodegenerative disease, sharing many features of other well-known neurodegenerative diseases. The relationships between neurodegenerative diseases and glaucoma have been widely investigated, showing positive relationships between the disorders. However, there are still conflicting results regarding whether glaucoma is a risk factor for the development of neurodegenerative disorders. In this study, using longitudinal data from a nationwide cohort, we demonstrated an increased risk of neurodegenerative disease development in OAG patients during a 10-year follow-up period compared with a control group. Subgroup analyses revealed that primary OAG patients had a significantly higher risk of developing AD, whereas a non-significant association was found for PD. The relationship between AD and OAG was stronger in older patients. There was no significant (*p* = 0.222) difference in patients younger than 60 years; in patients over 70 years old, the relationship was stronger than in younger patients and the statistical significance was increased (HR = 2.614, *p* < 0.001). To our knowledge, this is the first nationwide population-based cohort study from South Korea on the association between OAG and the risk of AD and PD.

Experimental and clinical studies have demonstrated a strong link between AD and primary OAG. While the presence of extracellular Aβ plaques and neurofibrillary tangles consisting of phosphorylated Tau protein is a characteristic feature of AD, many experimental studies have revealed increased levels of Aβ and Tau protein *in vivo* and *in vitro*. A recent study showed an increased Aβ level in the optic nerve and RGC layer in glaucomatous animal models^21,22^, and tauopathy was also detected in the glaucomatous retina^23^. A recent study observed these AD-like pathologies in the lateral geniculate nucleus and primary visual cortex in a chronic hypertensive glaucoma monkey model^24^. However, the results of epidemiological studies are still conflicting. Retrospective registry-based studies conducted in Denmark and the United States found no increased risk of developing AD in OAG patients^30,31,34^. Conversely, other studies found a positive correlation between AD and OAG and found that patients with AD had a higher prevalence of OAG than controls^35,36^. In recent studies from Taiwan, patients with OAG had a 1.5-fold increased chance of AD^37^, and the prevalence of AD in OAG patients was higher than in the control group, implying that OAG is a significant predictor of AD in elderly patients^29^. Our findings from the Korean population are consistent with these studies supporting the significant relationship between primary OAG and subsequent AD development.

PD is a long-term neurodegenerative disease of the central nervous system that mainly affects the motor system^13^ and is the second most common neurodegenerative disease after AD, affecting approximately seven million people globally^9^. Previously, abnormalities in visual function have been reported in PD patients^25^ and numerous studies have reported changes in the retinal nerve fiber layer thickness^26^. One study found that patients with PD had a higher prevalence of OAG^27^. Subsequently, two studies examined whether primary OAG is an early manifestation of PD: one found a small correlation but a significant increase in the risk of PD (adjusted HR = 1.23)^38^, and the other found that the prevalence of PD did not increase in OAG patients^29^. In this study, we also examined whether OAG is an early manifestation of PD in Koreans, but found no evidence for this. AD and PD share neuroinflammation as a common pathogenic mechanism and the retina is an extension of the brain. Therefore, the neuroinflammatory response in the brain may also occur in the retina and may lead to the development of glaucoma^39^. However, these diseases are caused by the deposition of different protein aggregates in specific anatomical areas and neuronal cell death in one or more populations of neurons. In AD, beta-amyloid and neurofibrillary tangles consisting of phosphorylated Tau protein accumulate in the hippocampal and cortical neurons, while in PD, Lewy bodies consisting of alpha-synuclein accumulate in the nigrostriatal neurons^14,39^. These pathogenic differences may have caused the discordant results in the AD and PD groups in our study. Additional studies are needed to address this issue.

The results of our subgroup analyses suggested particular associations between AD and OAG, which, to our knowledge, have not been reported. In the OAG group, the risk of developing AD during the 10-year period was higher in OAG patients older than 65 years of age compared with those younger than 65 years of age. This relationship implies that the two disorders are mainly related to the neurodegeneration associated with aging. There were also different associations for males and females, with female OAG patients having a greater risk of developing AD than male patients. This was consistent with a report that not only was OAG more prevalent in women but also that the incidence of AD was higher in elderly women than in men^40,41^. Moreover, OAG patients with diabetes mellitus or hyperlipidemia had higher risks of developing AD. Although still controversial, numerous studies have identified diabetes mellitus and hyperlipidemia as risk factors for both OAG and AD^7,42–45^. Conversely, metformin and statins, which are the most prescribed medications for diabetes mellitus and hyperlipidemia, respectively, have shown protective effects on the neurodegenerative changes in both glaucoma and AD^46–49^, making it more difficult to conclude the exact relationships between the two comorbidities and OAG or AD. Hypertension and ischemic stroke are also related to either OAG or AD^44,50–52^, although we found similar HR values for the subgroups with or without hypertension or ischemic stroke.

There are several limitations to consider when interpreting our results. First, due to the inherent nature of the data used in our analyses, the diagnoses of OAG, AD, PD, and other comorbidities were based entirely on the KCD code system, and may be less accurate than diagnoses obtained through a standardized diagnostic process. To ensure the diagnosis of OAG, we included those who satisfied three inclusion criteria as OAG patients: the diagnostic code for OAG; the code for visual field tests; and prescriptions of antiglaucoma drugs. We believe that the OAG subjects included in our study had high specificity. Second, it is possible that the OAG patients may have been underestimated because the data are based on hospital visits. The data do not include initial patients who did not experience symptoms and patients who do not visit the hospital for economic reasons. However, the degree of underestimation is limited, due to the low cost and high accessibility of the Korean medical system, a government-run healthcare system including nearly the entire population of South Korea. Therefore, it is unlikely that inaccuracy and underestimation of the diagnosis affected the statistical results. Third, our data include only data for the Korean population, and there may be differences in other ethnic groups. Studies from Asian ethnicity have reported a positive association between OAG and AD^28,29,37^. By contrast, studies conducted in Denmark, the United Kingdom, and the United States showed that this was not relevant^30,32,34^. Further studies are required to reveal specific differences in different ethnic groups.

In conclusion, this 10-year nationwide cohort study demonstrated that OAG was associated with an increased risk of developing AD compared with the risk in the general population. However, there was no positive association between OAG and PD. In addition, OAG patients older than 65 years of age or female OAG patients were more likely to develop AD than those under 65 years of age or male OAG patients. The results of this study have clinical implication on current understandings on OAG and further add to the evidences that OAG is a type of neurodegenerative disease. OAG patients should be followed closely for the subsequent development of symptoms related to AD, with neurological examinations for an early diagnosis of AD.

## Methods and Materials

### Database and study sample

We used data from the NHIS-NSC 2002–2013. The Korean National Health Insurance Service (KNHIS) is a mandatory single medical insurer system that provides universal healthcare coverage in the Republic of Korea, and all citizens are obligated to enroll in the system. The KNHIS developed the National Health Information Database (NHID), which contains personal demographic records to collect insurance premium and subscription data for reimbursement. The NHIS-NSC sampled 1,025,340 individuals from among 46,605,433 individuals in the NHID in 2002 and followed them until 2013^53^. The study design was approved by the Institutional Review Board of Soonchunhyang University Bucheon Hospital, Gyeonggi-do, Korea. This study adhered to the tenets of the Declaration of Helsinki, and the need for written informed consent was waived.

### Selection of glaucoma cases and controls

The index OAG cases were defined as patients who met all three of the following criteria: (1) diagnosed with primary OAG (Korean Classification of Diseases [KCD] code H401) as the main diagnosis code; (2) having a visual field test code (E6691); and (3) prescribed glaucoma medication. To exclude chronic OAG patients, we included patients who were diagnosed with OAG from 2004 to 2008. As a result, there were 1,587 index OAG cases. We excluded 118 patients who were diagnosed with AD (KCD codes F009, G300, G301, G308, and G309) or PD (code G20) before the OAG diagnosis, and ultimately enrolled 1,469 subjects.

Control subjects were selected from individuals who did not meet the OAG case criteria each year in a 1:5 ratio to glaucoma patients using propensity score with nearest neighbor matching^54^. Ultimately, 7,345 control subjects were included. Propensity scores were calculated using sociodemographic parameters, including age group (≤49, 50–59, 60–69, 70–79, ≥80), sex, income (≤40^th^, 41^st^–70^th^, ≥71^st^ percentiles), residential area (urban, metropolitan cities, and cities, rural, all other areas), and Charlson comorbidity index (CCI; score <3, ≥3). We used the CCI to determine whether the subject visited medical facilities frequently^55^.

### Outcomes and comorbidities

In the matched populations, we compared the incidence of AD and PD. Comorbid conditions included hypertension (codes I10–I15), diabetes (codes E10–E14), hyperlipidemia (codes E78.0–E78.5), and ischemic stroke (code I61) that were diagnosed between 2002 and 2008.

### Statistical analysis

The baseline characteristics are shown as the mean and standard deviation or number and percentage. The chi-square test was used to compare the groups. Multivariate Cox proportional hazard regression analysis was performed to calculate the HR with the 95% confidence interval (CI) for estimating the incidence of AD or PD in the OAG patients. Three models were used to demonstrate HRs for developing subsequent AD or PD, which included crude model, minimal adjusted model 1 (adjusted for hypertension, diabetes mellitus, hyperlipidemia and ischemic stroke) and fully adjusted model 2 (adjusted for age, sex, residential area, income, CCI, hypertension, diabetes mellitus, hyperlipidemia and ischemic stroke). Subgroup analyses were conducted for age group (≥65 and <65), sex, and comorbidities. Survival curves for developing AD or PD were generated. All statistical analyses were conducted using SAS 9.4 (SAS Institute, Cary, NC, USA) and R 3.4.1 (R Project for Statistical Computing, Vienna, Austria).

## Electronic supplementary material

Supplementary Information 
