# Supplementary material for: Association between Open-Angle Glaucoma and the Risks of Alzheimer’s and Parkinson’s Diseases in South Korea: A 10-year Nationwide Cohort Study
Source: Sci Rep. 2018 Jul 24;8:11161. doi: 10.1038/s41598-018-29557-6 (PMC6057948; doi:10.1038/s41598-018-29557-6)
Supplement: Supplementary file 1 — Supplementary Information [file 41598_2018_29557_MOESM1_ESM.doc]

# Association between Open-Angle Glaucoma and the Risks of Alzheimer’s and Parkinson’s Diseases in South Korea: A 10-year Nationwide Cohort Study

Jong Youn Moon^1,2#^, Hyung Jun Kim^3#^, Yoon Hyung Park^1^, Tae Kwann Park^4^, Eun-Cheol Park^2^, Chan Yun Kim^5^, Si Hyung Lee^4*^.

^#^: These authors contributed equally to this work.

*: corresponding author

^1^Department of Preventive Medicine, Soonchunhyang University, College of Medicine, Cheonan, Republic of Korea; ^2^Institute of Health Services Research, Yonsei University, College of Medicine, Seoul, Korea; ^3^Department of Neurology, Soonchunhyang University Hospital Bucheon, Bucheon, Republic of Korea; ^4^Department of Ophthalmology, Soonchunhyang University Hospital Bucheon, Bucheon, Republic of Korea; ^5^Institute of Vision Research, Department of Ophthalmology, Severance Hospital, Yonsei University, College of Medicine, Seoul, Korea.

Supplementary table 1. Hazard ratios for Alzheimer's disease or Parkinson's disease in multivariable Cox regression analysis (Model 1)

| **Variables** |  | **AD** | | | **PD** | | |
| --- | --- | --- | --- | --- | --- | --- | --- |
|  |  | **Hazard ratio (95% CI)** | | **p-value** | **Hazard ratio (95% CI)** | | **p-value** |
| OAG | No | 1.000 (reference) |  |  | 1.000 (reference) |  |  |
|  | Yes | 1.421 | (1.199 -1.684) | <0.001 | 0.984 | (0.612 - 1.582) | 0.946 |
| Hypertension | No | 1.000 (reference) |  |  | 1.000 (reference) |  |  |
|  | Yes | 2.523 | (1.862 – 3.420) | <0.001 | 3.054 | (1.427 – 6.535) | 0.004 |
| Diabetes | No | 1.000 (reference) |  |  | 1.000 (reference) |  |  |
|  | Yes | 1.794 | (1.438 – 2.237) | <0.001 | 1.331 | (0.777 – 2.277) | 0.298 |
| Hyperlipidemia | No | 1.000 (reference) |  |  | 1.000 (reference) |  |  |
|  | Yes | 1.016 | (0.814 - 1.268) | 0.890 | 0.873 | (0.513 – 1.487) | 0.618 |
| Ischemic stroke | No | 1.000 (reference) |  |  | 1.000 (reference) |  |  |
|  | Yes | 4.851 | (4.115 – 5.718) | <0.001 | 3.311 | (2.189 – 5.008) | <0.001 |

**Supplementary table 2. Hazard ratios for Alzheimer's disease or Parkinson's dise**ase in multivariable Cox regression analysis (Model 2)

| **Variables** |  | **AD** | | | **PD** | | |
| --- | --- | --- | --- | --- | --- | --- | --- |
|  |  | **Hazard ratio (95% CI)** | | **p-value** | **Hazard ratio (95% CI)** | | **p-value** |
| OAG | No | 1.000 (reference) |  |  | 1.000 (reference) |  |  |
|  | Yes | 1.403 | (1.180 -1.669) | <0.001 | 0.995 | (0.620 - 1.595) | 0.983 |
| Hypertension | No | 1.000 (reference) |  |  | 1.000 (reference) |  |  |
|  | Yes | 1.095 | (0.817 - 1.468) | 0.543 | 1.596 | (0.752 - 3.390) | 0.223 |
| Diabetes | No | 1.000 (reference) |  |  | 1.000 (reference) |  |  |
|  | Yes | 1.157 | (0.932 - 1.437) | 0.185 | 0.922 | (0.534 - 1.591) | 0.851 |
| Hyperlipidemia | No | 1.000 (reference) |  |  | 1.000 (reference) |  |  |
|  | Yes | 1.105 | (0.896 - 1.362) | 0.352 | 0.815 | (0.485 - 1.371) | 0.441 |
| Ischemic stroke | No | 1.000 (reference) |  |  | 1.000 (reference) |  |  |
|  | Yes | 2.778 | (2.778 - 3.279) | <0.001 | 2.262 | (1.446 - 3.539) | <0.001 |
| Age group | ≤ 49 | 1.000 (reference) |  |  | 1.000 (reference) |  |  |
|  | 50-59 | 33.313 | (4.517 – 245.712) | <0.001 | 3.924 | (0.824 – 18.690) | 0.086 |
|  | 60-69 | 90.006 | (12.352 – 655.838) | <0.001 | 11.845 | (2.663 – 52.678) | 0.001 |
|  | 70-79 | 263.719 | (36.200 – 1921.185) | <0.001 | 14.148 | (3.084 – 64.901) | <0.001 |
|  | ≥ 80 | 339.081 | (46.199 – 2488.719) | <0.001 | 12.328 | (2.472 – 61.480) | <0.001 |
| Sex | Male | 1.000 (reference) |  |  | 1.000 (reference) |  |  |
|  | Female | 1.239 | (1.068 - 1.437) | 0.005 | 1.166 | (0.799 - 1.702) | 0.426 |
| Residential area | Metropolitan | 1.000 (reference) |  |  | 1.000 (reference) |  |  |
|  | City | 0.977 | (0.738 - 1.294) | 0.873 | 2.215 | (0.803 - 6.108) | 0.124 |
|  | Rural | 1.112 | (0.844 - 1.465) | 0.451 | 2.476 | (0.903 - 6.791) | 0.078 |
| Income | High | 1.000 (reference) |  |  | 1.000 (reference) |  |  |
|  | Milddle | 0.831 | (0.690 – 1.002) | 0.052 | 0.831 | (0.525 - 1.317) | 0.432 |
|  | Low | 1.238 | (1.016 - 1.507) | 0.034 | 0.991 | (0.596 - 1.648) | 0.971 |
| CCI | ≤3 | 1.000 (reference) |  |  | 1.000 (reference) |  |  |
|  | >3 | 1.906 | (1.623 - 2.238) | <0.001 | 1.809 | (1.150 - 2.847) | 0.010 |

OAG = open angle glaucoma; AD = Alzheimer’s disease; PD = Parkinson’s disease; CCI = Charlson comorbidity index.
